# Supplementary material for: Preparing local strain patterns in graphene by atomic force microscope based indentation
Source: Sci Rep. 2017 Jun 8;7:3035. doi: 10.1038/s41598-017-03332-5 (PMC5465061; doi:10.1038/s41598-017-03332-5)
Supplement: Supplementary file 1 — Supplementary information [file 41598_2017_3332_MOESM1_ESM.doc]

**Supplementary information for:**

**Preparing local strain patterns in graphene by atomic force microscope based indentation**

Péter Nemes – Incze^1,*^ , Gergő Kukucska^2^, János Koltai^2^, Jenő Kürti^2^, Chanyong Hwang^3^, Levente Tapasztó^1^, and László P. Biró^4^

1 Centre for Energy Research, Institute of Technical Physics and Materials Science, Nanotechnology Department, 2D NanoFab ERC Research Group, Budapest 1525, POB 49, Hungary

2 Department of Biological Physics, Eötvös University (ELTE), Pázmány Péter sétány 1/A, 1117 Budapest, Hungary

3 Korea Research Institute of Standards and Science, Center for Nanometrology, Daejeon 305-340, Republic of Korea

4 Centre for Energy Research, Institute of Technical Physics and Materials Science, Nanotechnology Department, Budapest 1525, POB 49, Hungary

* corresponding author email: nemes.incze.peter@energia.mta.hu

**Graphene damage during patterning:**

If graphene is pushed into the substrate by more than 1.5-2 nm the chances of rupture are significant. In the example below, the line at the right edge of the pattern has been ruptured. The rupture shows up in the AFM topography image as a roughening of the indent line edge. Significant perturbation of the 2D peak position Raman map can also be observed.

§


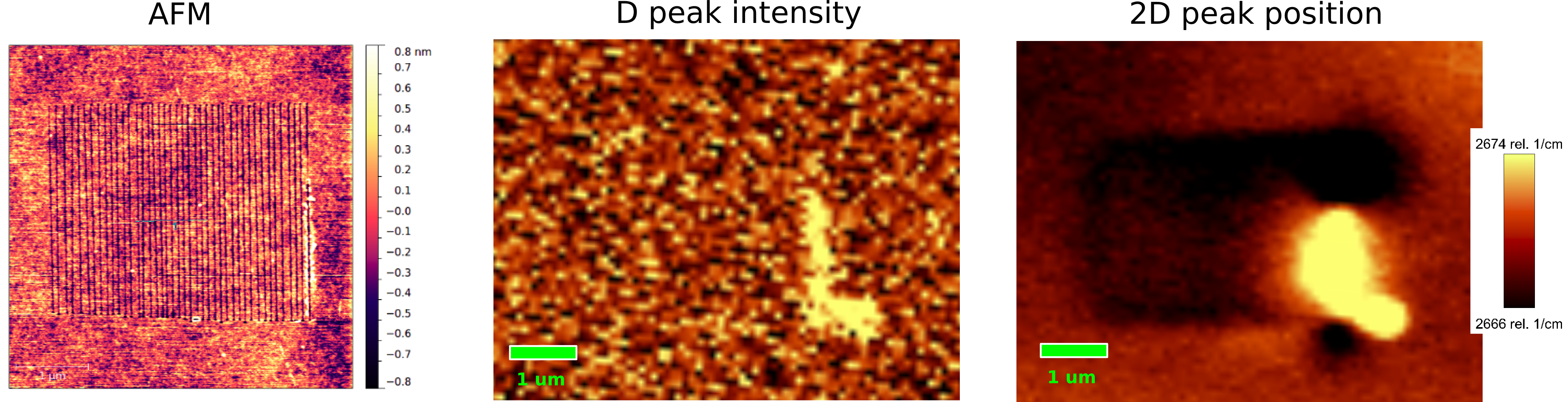
**Figure S1.** While preparing the rightmost indent line, the graphene has been torn. This is visible in the AFM topography image, as well as the 2D peak position signal, which gets strongly perturbed near the defect. At the same time, a significant D peak is observed.

**Stability of strain patterns over time:**

Samples did not show significant changes in the strain measured via Raman over periods of days, weeks or months. Significant changes to the strain and sample doping have been observed only if the laser power was not kept below 1 mW.


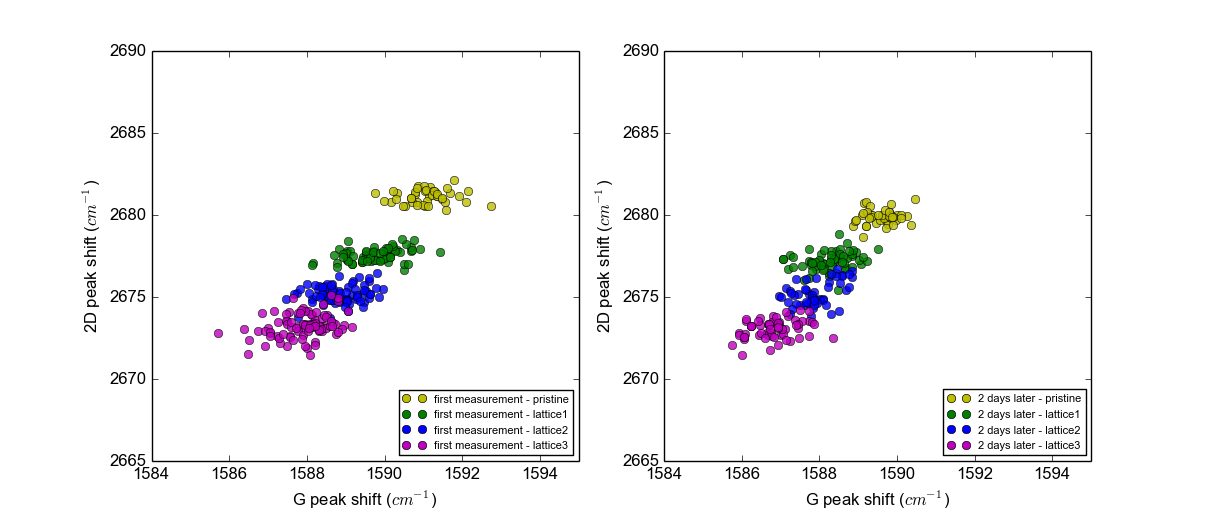
**Figure S2.** Same dataset as in Figure 1d in the main text. Spectra on the right were taken 2 days after the spectra in the left on the same indent patterns.

**Using PMMA as substrate:**


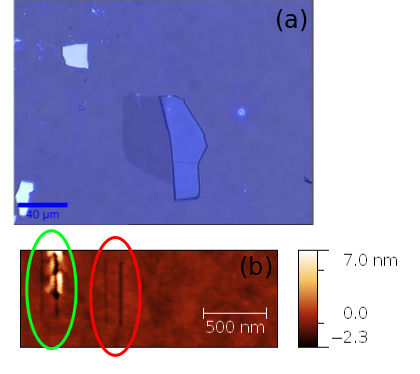


***Figure S3****. (a) Optical microscope image of graphene on PMMA. (b) AFM image of indentation of graphene on PMMA. Red ellipse: indentation lines, with varying indent depth. Green ellipse: torn graphene due to high indent depth.*

**AFM images to Figure 1:**

In order to confirm the indentation depth of the structures shown in Figure 1 of the main text. We include here the AFM images of all the indentation patterns.


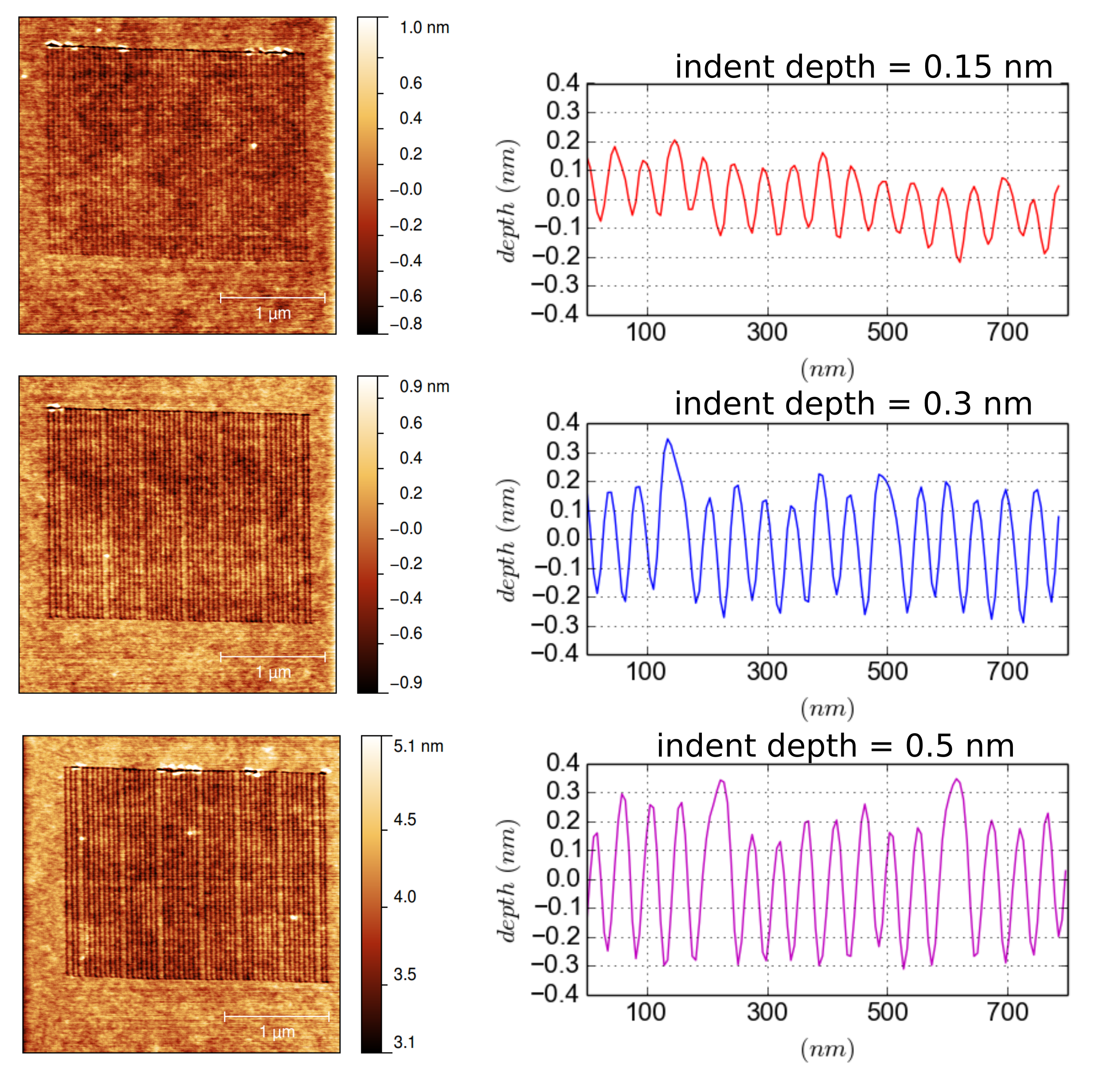


***Figure S4****. AFM topography images (left column) and height sections (right column) of the line patterns in Figure 1c of the main text.*

**Strain distribution in a Gaussian depression**

Below we visualize the magnitude of the atomic displacements within the Gaussian depression of a 1nm depth and 7 nm spread, used to calculate the pseudomagnetic field distribution in Figure 3c of the main text. The atomic distances are plotted in the three nearest neighbor directions as a color scale on the carbon atoms and bonds. The maximum bond stretching is found to be 0.36%, larger than the strain measured within the ~500 nm diameter laser spot of 0.17%. This is to be expected, since the information gathered in the Raman signal is an average of sample areas of higher and lower strain.


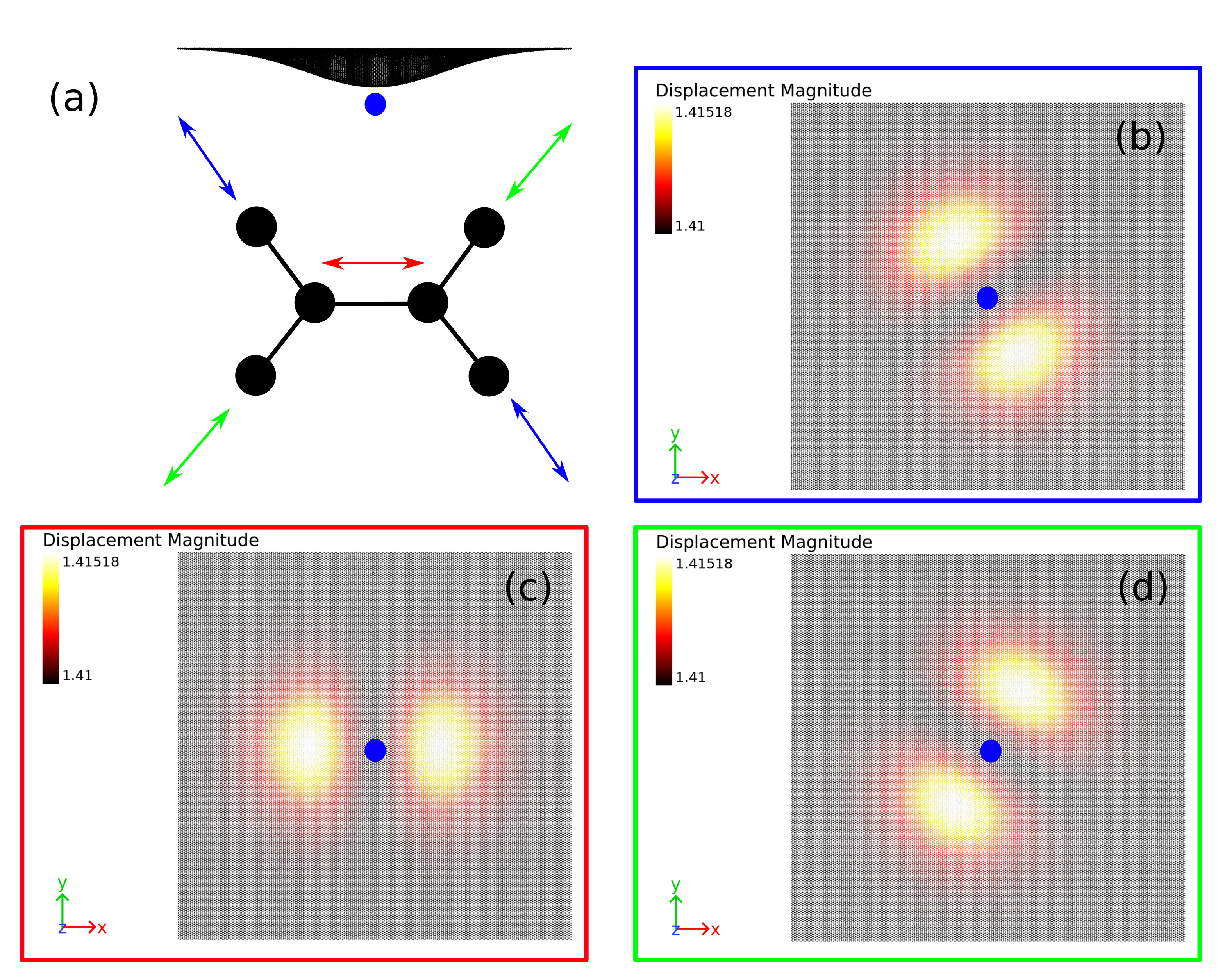


***Figure S5****. Variation of the atom displacement magnitudes (bond stretching) for the Gaussian bump in Figure 3c. (a) crystallographic orientation of the graphene lattice and colored arrows showing the three directions for which the displacement magnitude is plotted. Side view of the Gaussian depression.(b-d) The atomic displacement magnitude is plotted in boxes with edge color corresponding to the arrow colors in a. Blue dots show the position of the Gaussian minimum in all images.*


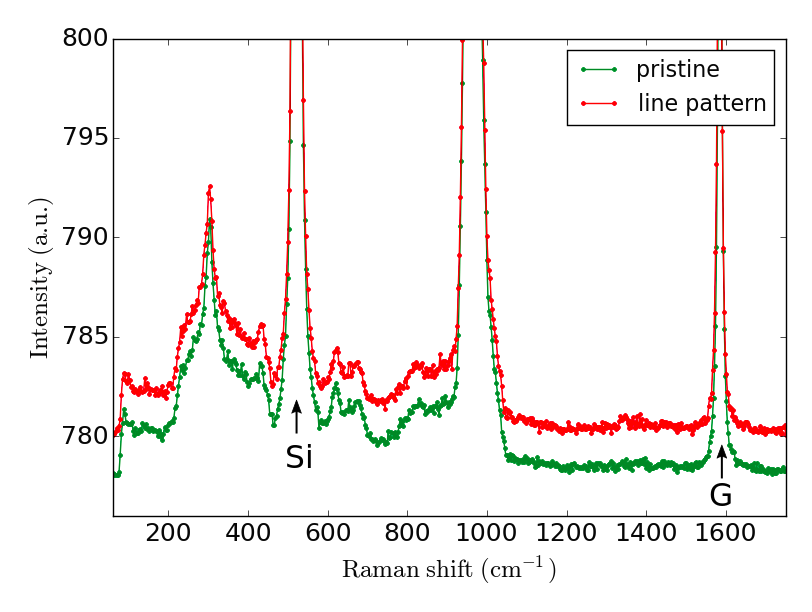


***Figure S6****. Raman spectra showing the peaks of the Si/SiO_2_ surface. No changes are seen within the substrate peaks due to AFM indentation. The green spectrum is of the pristine area next to a line pattern (red spectrum). The line pattern is the one presented in Figure 2a,c of the main text. The spectra are offset for clarity.*
